# The Statement Issued by the Central Committee for State Registration in London

**Published:** 1916-12-09

**Authors:** 


### THE STATEMENT ISSUED BY THE CENTRAL COMMITTEE FOR STATE REGISTRATION IN LONDON.

### The Committee's Attitude Defined.

At its meeting on September 28 the Central Committee resolved to inform the Council of the College that if four amendments which were set out were not adopted by the College the Committee would not continue negotiations, but proceed with its own Bill. This was without prejudice to the further consideration of minor amendments.

The four amendments to which the Central Committee attached so much importance were those relating to the constitution of the Provisional and Permanent Nursing Councils, to the registration of nurses in practice at the time of the passing of the Act, and to the qualifications of nurses after the passing of the Act. The Committee was of opinion that there was no guarantee that either of the Nursing Councils formed in the manner proposed by the Council of the College of Nursing would be representative of the interests concerned, and therefore desired to have it plainly stated in the Bill what bodies should appoint, or elect, the members of the Provisional and Permanent Councils.

In respect of the other two points, the Central Committee was of opinion that the clause in the Bill of the College of Nursing relating to the nurses in practice at the passing of the Act was not satisfactory, in that the conditions for admission to the Register are not explicitly stated, and that the clause which specified the conditions of registration of nurses after the passing of the Act was too vague, as it did not enforce any hospital training or examination before registration.

There is, moreover, the fundamental objection in the Bill of the College that a voluntary College is to be associated with a Statutory Council. It is one thing for the College of Nursing to be represented on a Statutory General Nursing Council; it is quite another for the Council of the College to *be* the General Nursing Council.

As in the communication received from the College it did not signify its agreement to the amendments considered essential, the Central Committee at its meeting on October 21 reaffirmed its intention, by a majority of 20 to 2, to proceed with its own Bill, in connection with which it considered and agreed to certain amendments in order to bring it up to date, and reaffirmed the fundamental principles as follows:

1. That provision should be made for a Statutory General Nursing Council to regulate the qualifications of trained nurses and provide for their registration.
2. For direct representation of the nursing profession on the General Nursing Council, and the insertion in the Bill of the authorities empowered to nominate the Council.
3. For a three-years' term of grace, after the passing of the Act, for the registration of nurses in practice—who hold a certificate of training or produce evidence of training satisfactory to the Council—without further examination.
4. For the provision at the expiration of the said term of grace that a nurse must have had not less than three years' training under a definite curriculum prescribed by the Council in the wards of a hospital, or of hospitals, approved by the Council, and that such person must have passed such examination as the Council may prescribe.

### MAINLY HISTORICAL.

The following statement has been prepared on the instruction of the Central Committee by its Executive Committee concerning the negotiations which, unfortunately, have not resulted in agreement between the Central Committee for the State Registration of Nurses and the College of Nursing, Ltd., negotiations entered into with the object of drafting a conjoint Nurses' Registration Bill.

The question of the State registration of trained nurses has been before the public for the past quarter of a century, and, upon the initiation of the Society for the State Registration of Trained Nurses, a Bill was drafted which was introduced into Parliament in 1904, and in every subsequent session up to 1909.

In 1909 there were three Bills for this reform before Parliament, promoted respectively by the Society for the State Registration of Trained Nurses, the Royal British Nurses' Association, and the Association for Promoting the Registration of Nurses in Scotland.

In January 1910, upon the suggestion of the Society for the State Registration of Trained Nurses, a Conference was held in London with the object of drafting a Bill which would be acceptable to the three bodies which at that time had Bills before Parliament, and to a number of Societies interested in the subject of State registration of nurses.

At this Conference the Bill promoted by the Society for the State Registration of Trained Nurses, which Lord Ampthill had carried through the House of Lords in 1908, was taken as a basis of negotiations. Lord Ampthill presided, and the following Societies were represented:

The Royal British Nurses' Association, the Matrons' Council of Great Britain and Ireland, the Society for the State Registration of Trained Nurses, the Fever Nurses' Association, the Association for Promoting the Registration of Nurses in Scotland, the Scottish Nurses' Association, the Irish Nurses' Association, and the British Medical Association.

At this Conference a permanent Committee—the Central Committee for the State Registration of Nurses—consisting of five delegates from each of the above-mentioned Societies, was formed—to which the National Union of Trained Nurses has since been affiliated—for the purpose of securing united action in regard to State registration until a satisfactory law was passed by Parliament.

As a result of the Conference a Bill was agreed upon which received the support of all the Societies represented.

The Nurses' Registration Bill, above referred to, was introduced into the House of Commons by Mr. (now Sir Ronald) Munro Ferguson in 1910, and at every subsequent session of the House until 1914.

As it was persistently blocked, and the Government did not allot time for its consideration, it did not reach a second reading.

In March 1914 the Bill was introduced by Dr. Chapple under the Ten Minutes' Rule, and, upon a division being demanded, leave to bring in the Bill was given by a majority of 229.

When war broke out the Prime Minister debarred the introduction by private members of contested Bills into the House of Commons on subjects unconnected with the war, and the Bill of the Central Committee was therefore not introduced in 1915. The Committee realised also that the care of the sick and wounded would engage the attention and energy of the medical and nursing professions to the almost total exclusion of other subjects.

But on December 30, 1915, the Honble. Arthur Stanley, M.P., Chairman of the Joint War Committee of the British Red Cross Society and the Order of St. John, addressed a letter to the Chairmen of the Committees of Management of the principal hospitals and infirmaries in the United Kingdom, in which he advocated the establishment of a College of Nursing.

In this letter Mr. Stanley stated that "there is no unanimous feeling, either amongst those responsible for the training of nurses or amongst the nurses themselves, in favour of any system of State registration." and that his own view was "that, for the time at least, we must rely upon a voluntary scheme of co-operation amongst the Nurse Training Schools throughout the country."

In appealing to the chairmen and governors of leading hospitals, physicians and surgeons lecturing to nurses, the principals of Nurse Training Schools and of Nursing Associations, and other persons interested in the education of women to promote the College, Mr. Stanley failed to approach the Central Committee or any of the self-governing associations of nurses.

His proposition was that the College was to be registered, with its Memorandum and Articles of Association, at the Board of Trade, and that application should be made to the Board for its approval to omit the word "Limited" from the title of the Association. Notice was given, however, to the Board of Trade that certain trained nurses' associations intended to oppose the application in the event of its being made, as they had successfully opposed in 1905 a practically identical scheme which had been promoted, under the title of "The Incorporated Society for Promoting the Higher Education and Training of Nurses," primarily by some of the persons who were now promoting the College of Nursing.

The promoters of the College therefore decided to register with the Board of Trade as a "Company Limited by Guarantee and not having a share capital." The medical and nursing professions had, therefore, no opportunity of representing their objections to the Board of Trade as they did in 1905.

Meanwhile, as one of the objects of the College, as stated in the Memorandum of Association, was to promote Bills in Parliament for any object connected with the interests of the nursing profession and, in particular, with the education or organisation and protection of nurses, and for their recognition by the State, the founding of the College was a matter of peculiar interest to the Central Committee, which had been formed for the furtherance of these very objects.

At a meeting held on January 15, 1916, the Central Committee resolved to communicate with Mr. Stanley and his advisers, and ask Mr. Stanley to receive representatives of the Central Committee, and to afford them further information concerning his proposed scheme.

In consequence, two meetings of representatives of the Central Committee and the College were held on March 2 and on March 24. The latter meeting was of some importance, because the following resolution was then passed with only two dissentients: "That this meeting affirms as the basis of any agreement the necessity of (1) State Registration. (2) uniform curriculum, and (3) a one-portal examination, after such period of training as may be found desirable." the promoters of the College presumably having become convinced that a system of voluntary registration would not receive the support of the nursing profession, a fact well known to those persons who had been promoting this reform for many years.

At this meeting Mr. Stanley also announced that the promoters of the College had nominated certain ladies and gentlemen to form the first Council of the College.

At its meeting on April 13 the Central Committee for the State Registration of Nurses considered a letter from Mr. Stanley inviting the Committee to appoint nine delegates to meet a Sub-Committee of the Council of the College of Nursing, consisting of an equal number, "with the object of coming to an agreement upon the terms of a Bill to be brought before Parliament as an agreed Bill at as early a date as possible." To this the Committee agreed, provided that its Bill was taken as the draft to be considered. This proviso, however, was not carried out, but subsequently the College drafted a Bill, and upon the promise of Mr. Stanley that the Central Committee should, together with the College of Nursing, be inserted in the Bill as appointing an equal number of representatives on the Provisional Council—thus securing the direct representation of the organised nurses' societies on the Council which would frame the rules and regulations to which the registered nurses would have to conform—meetings of accredited representatives of the Central Committee and of the College took place. At these meetings the College representatives agreed to incorporate several other important provisions which the Central Committee considered essential for the protection of the nursing profession, and the Central Committee, in its desire to arrive at agreement with the College, went to the extreme limit of concessions compatible with the future safety of the nursing profession.

At the meeting of the Central Committee on June 22, in the Draft Bill of the College under consideration the Central Committee was included as a nominating body on the Provisional Council, thus providing for the direct representation of the constituent nurses' organisations formed to secure State Registration of Nurses, but, in the draft considered on July 13, the Central Committee had been eliminated, presumably by the Council of the College, and a proposal substituted that the first Council should be composed of forty-five people to be mentioned by name in the Bill, a breach of agreement which deprives the nursing profession of *direct* representation on their own governing body.

⁂ The above statements are critically examined in an article on p. 205.

For Matrons' and Sisters' Appointments, see p. vi. For "Trained Nurses and the Anglo-French Committee," see p. 209. For the Central Committee's "Statement," see p. 207.

